# Involvement of MicroRNAs in the Hypersensitive Response of Capsicum Plants to the Capsicum Chlorosis Virus at Elevated Temperatures

**DOI:** 10.3390/pathogens13090745

**Published:** 2024-08-31

**Authors:** Wei-An Tsai, Christopher A. Brosnan, Neena Mitter, Ralf G. Dietzgen

**Affiliations:** Centre for Horticultural Science, Queensland Alliance for Agriculture and Food Innovation, The University of Queensland, St. Lucia, QLD 4072, Australia

**Keywords:** capsicum chlorosis orthotospovirus, resistance-breaking, small RNA libraries, high-throughput sequencing

## Abstract

The orthotospovirus capsicum chlorosis virus (CaCV) is an important pathogen affecting capsicum plants. Elevated temperatures may affect disease progression and pose a potential challenge to capsicum production. To date, CaCV-resistant capsicum breeding lines have been established; however, the impact of an elevated temperature of 35 °C on this genetic resistance remains unexplored. Thus, this study aimed to investigate how high temperature (HT) influences the response of CaCV-resistant capsicum to the virus. Phenotypic analysis revealed a compromised resistance in capsicum plants grown at HT, with systemic necrotic spots appearing in 8 out of 14 CaCV-infected plants. Molecular analysis through next-generation sequencing identified 105 known and 83 novel microRNAs (miRNAs) in CaCV-resistant capsicum plants. Gene ontology revealed that phenylpropanoid and lignin metabolic processes, regulated by Can-miR408a and Can- miR397, are likely involved in elevated-temperature-mediated resistance-breaking responses. Additionally, real-time PCR validated an upregulation of Can-miR408a and Can-miR397 by CaCV infection at HT; however, only the *Laccase 4* transcript, targeted by Can-miR397, showed a tendency of negative correlation with this miRNA. Overall, this study provides the first molecular insights into how elevated temperature affects CaCV resistance in capsicum plants and reveals the potential role of miRNA in temperature-sensitive tospovirus resistance.

## 1. Introduction

Capsicum or pepper, belonging to the genus *Capsicum* and family Solanaceae, is a nutritionally important vegetable that originated in Central and South America, the Caribbean, and Mexico, with at least 27 reported species [1,2]. Capsicum production has increased due to strong demand and culinary use of its fruit [3,4]; however, its growth is frequently threatened by different abiotic and biotic stresses and can be affected by those stresses simultaneously [3,4,5,6]. Therefore, it is of increasing importance to study the impact of combined stresses on capsicum plants, such as virus infection and high temperature.

Capsicum chlorosis virus (CaCV) is a serious pathogen that infects not only capsicum but also chili, tomato, pineapple, and peanut crops [7,8,9]. The virus has been reported in Australia, China, Greece, India, Iran, Taiwan, Thailand, and the USA [10]. In southern China, CaCV is considered a significant disease in peanuts, with incidence reaching up to 20% and causing noticeable yield losses, particularly when plants are infected at early growing stages [10,11]. In India, the virus affects the production of chili peppers, with disease incidences over 20% reported [10,12]. In Australia, the emergence of CaCV has been reported in large commercial capsicum production areas, including Bundaberg and the northern dry tropics of Queensland [9]. The typical CaCV symptoms on capsicum plants include stunting, marginal and interveinal chlorosis and leaf deformation on emerging leaves, and concentric chlorotic or necrotic lesions on mature leaves [9,13]. Recently, CaCV-resistant advanced breeding lines (PI 290972 × *C. annuum* cv. Mazurka and cv. Warlock inbred lines) were selected by the Queensland Department of Agriculture and Fisheries (DAF) breeding program [13,14]. CaCV is taxonomically classified in the species *Capsicum chlorosis orthotospovirus* in the genus *Orthotospovirus*, family *Tospoviridae*, order *Bunyavirales* [15]. The genome of this orthotospovirus consists of three segments of single-stranded RNA (ssRNA). Among them, the large (L) segment is of negative polarity and encodes the RNA-dependent RNA polymerase (RdRp), while both medium (M) and small (S) segments are of ambisense coding polarity with viral movement protein (NSm) and glycoproteins (Gn/Gc) encoded on the M segment, and a viral silencing suppresser (NSs) and nucleocapsid protein (N) encoded on the S segment [13,16,17].

The progression of virus diseases in plants is significantly affected by temperature since both virus propagation and plant development are temperature-dependent [18]. Given current concerns about global warming, elevated temperatures have been recognized as an important climate-changing variable impacting plant–virus interactions [19,20,21]. To date, evidence shows that exposure to elevated temperatures suppresses effector-triggered immunity (ETI)-mediated plant resistance [22,23,24]. ETI is a robust plant defense response triggered by recognizing pathogen effector molecules through nucleotide-binding leucine-rich repeat (NLR) proteins [25]. Typical ETI responses are associated with calcium production, salicylic acid (SA) accumulation, and a burst of oxidative reactive oxygen species (ROS). Furthermore, these plant responses result in two typical manifestations of disease resistance, hypersensitive response (HR) and systemic acquired resistance (SAR) [25]. Examples of temperature-mediated breaking of disease resistance include the potato virus Y (PVY)–potato and tomato spotted wilt virus (TSWV)–capsicum pathosystems [24,26,27,28,29]. In these pathosystems, virus-resistant plant varieties, carrying resistance (R) genes, are unable to mount effective ETI at elevated temperatures. The capsicum *Tsw* gene and potato R gene, *Ny-DG*—which confer resistance to TSWV and PVY, respectively—are compromised at 32 °C or above 28 °C [26,29].

MicroRNAs (miRNAs), which are post-transcriptional regulators, are found in most eukaryotes [30]. In plants, miRNAs are conserved regulators of plant developmental processes and stress responses [31,32]. The 20–24 nucleotide (nt) miRNAs originate from miRNA genes (MIR). These genes are transcribed by RNA polymerase II (Pol II) into a stem–loop structure, named primary (pri)-miRNAs. Pri-miRNAs are processed by DICER-LIKE 1 (DCL1) and several assisting proteins to form the precursor (pre)-miRNAs and mature miRNA duplex. At the last step of this pathway, the guide strand of the miRNA duplex is loaded onto specific ARGONAUTE (AGO) proteins for target mRNA cleavage or translational inhibition [33]. MiRNAs are essential elements affecting the plant–virus arms race [34,35]. Several miRNAs have been reported to act as regulators to support plant antiviral resistance [36,37]. For example, the expression of miR6019/miR6020, which downregulates expression of the NLR receptor N gene in tobacco plants, is reduced, allowing effective induction of NLR-mediated ETI during tobacco mosaic virus (TMV) infection [37]. During infection of tomato leaf curl New Delhi virus, Sly-miR159 appears to be downregulated, which increases the expression of its target *SlMyB33* and the downstream R gene *SlSw5a* in resistant tomatoes. This modulation enhances the plant defense against the virus by triggering HR [38]. Conversely, some miRNAs act as regulators that are favorable for virus infections, like miR319 [39,40]. The expression of miR319 in rice plants is induced by rice ragged stunt virus infections. This leads to a suppression of jasmonic acid (JA)-mediated defenses, which results in an increased susceptibility of rice plants to virus disease [39].

Although the interplay between plant miRNAs and negative-sense RNA viruses has been explored [41], the role of miRNAs in capsicum resistance to CaCV at different temperatures remains unknown. Therefore, this study investigated the effects of high temperature on capsicum resistance to CaCV through phenotypic and miRNA analysis. Small RNA (sRNA) high-throughput sequencing (HTS) was used for a systemic comparative analysis of differentially expressed miRNAs in plants challenged with or without CaCV at ambient (25 °C) and elevated temperatures (35 °C/30 °C), elucidating miRNA-related underlying mechanisms involved in the capsicum–CaCV pathosystem.

## 2. Materials and Methods

### 2.1. Plants and Growth Conditions

CaCV-resistant capsicum plants from the third backcross generation of *Capsicum chinense* PI 290972 × commercial CaCV-susceptible *C. annuum* cultivars [14] were grown in a temperature-controlled glasshouse compartment at an ambient temperature (AT) of approximately 25 °C. Seedlings aged four weeks with two true leaves were mechanically inoculated with CaCV isolate QLD 3432 [17]. Inoculum was prepared by grinding fresh CaCV-infected symptomatic capsicum leaves of the susceptible cultivar Yolo Wonder in 10 mM phosphate buffer, pH 7.6, containing freshly added 20 mM sodium sulphite with a mortar and pestle. Mock treatment used buffer only. Inoculum and buffer were rub-inoculated onto the carborundum-dusted first two leaves and two cotyledons. Subsequently, half of each plant group was transferred to a growth cabinet at high temperature (HT) of 35 °C/30 °C (16 h day/8 h night), light intensity of 230 μmol·m^−2^·s^−1^, and relative humidity of 60%.

### 2.2. RNA Isolation, Small RNA Library Construction. and Sequencing

Capsicum plants, treated under four conditions, were used for sRNA library construction: mock-inoculated plants grown at AT (‘AM’ hereafter); CaCV-inoculated plants grown at AT (‘AV’ hereafter); mock-inoculated plants grown at HT (‘HM’ hereafter); and CaCV-inoculated plants grown at HT (‘HV’ hereafter). Systemic leaves from three individual plants, each grown in one of those four conditions, were collected at 10 dpi. For each biological replicate, three leaf disks from the third leaf from the top were sampled. Total RNA from sample lysates in lysis buffer (ISOLATE II RNA Plant Kit, Bioline, London, UK) was purified by Direct-zol RNA Miniprep kit (Zymo Research, Irvine, CA, USA) according to the manufacturer’s instructions. On-column DNase I treatment was performed using the same kit. Total RNA (2–3 µg per sample) was sent to the Australian Genome Research Facility (AGRF, Melbourne, VIC, Australia) for sRNA library construction and HTS. The sRNA libraries were prepared using NEBNext^®^ Small RNA Library Prep Set (New England Biolabs, Ipswich, MA, USA) for Illumina^®^ following the manufacturer’s instructions. Single-end 50 bp sequencing was performed on an Illumina NOVASEQ 6000, AGRF, Melbourne, VIC, Australia.

### 2.3. Small RNA Sequencing Data Pre-Processing and microRNA Identification

Raw reads generated by sRNA sequencing were pre-processed using the source code of iwa software (https://github.com/cma2015/iwa-miRNA/tree/master/Source_code/modulei, accessed on 21 January 2021) [42]. First, the FASTX-Toolkit v0.0.14 (http://hannonlab.cshl.edu/fastx_toolkit, accessed on 31 January 2021) was used to trim low-quality reads and adaptor (NEBnext: AGATCGGAAGAG). The threshold for low-quality read trimming was set as a minimum quality score of 20, with more than 80 percent of bases covered. Second, length_cutoff.sh script from iwa software was used to remove the reads with lengths <18 nt or >26 nt. Subsequently, all the length-limited reads were aligned against the *C. annuum* (CM334) reference genome v1.6 (http://peppergenome.snu.ac.kr/download.php, accessed on 14 January 2021) using Bowtie v1.2.2 with parameters -v1 –best –strata -m20 [43]. The mapped reads were used for quantification of capsicum miRNAs, and the reads that failed to map to the capsicum genome were used for CaCV viral siRNA (vsiRNA) quantification.

The MiRNA Compilation and MiRNA Selection modules in iwa software were used to identify the putative known and novel miRNAs [42]. Raw reads were uploaded into iwa software and were pre-processed by the same parameters mentioned above. In the MiRNA Compilation module, miRDeep-P2 was selected for miRNA prediction [44]. The PmiREN (http://www.pmiren.com/) [45], sRNAanno (http://www.plantsrnas.org/) [46], and PsRNA (https://plantsmallrnagenes.science.psu.edu/) [47] databases were used for annotating capsicum miRNAs. In the MiRNA Selection module, high-throughput criteria and the one-class support vector machine (SVM) classifier were applied to determine if tested miRNA candidates are real miRNAs [48,49]. InteractiVenn was then used to visualize all identified miRNAs using Venn diagrams [50]. After miRNA identification, all novel and known miRNAs that were identified in libraries across at least two conditions were used as the reference for miRNA quantification. The mapper and quantifier modules in MiRDeep2 v0.0.7 [51] were then used to collapse genome-mapped reads and count the reads of the identified miRNAs.

### 2.4. Analysis of Virus-Derived siRNAs

The genome of CaCV-Qld-3432, containing 8913 nt in the L segment, 4846 nt in the M segment, and 3944 nt in the S segment (GenBank accession numbers KM589495, KM589494, KM589493) [17], was used for sRNA alignment. The profile of 21-nucleotide, 22-nucleotide, and 24-nucleotide vsiRNA normalized read counts was obtained using SCRAM aligner module (https://sfletc.github.io/scram/). Subsequently, the SCRAM plotting module was used to show the read coverage across CaCV reference genome segments [52].

### 2.5. Differential Expression Analysis of miRNAs

Differentially expressed (DE) miRNAs in the two treatments were analyzed using DEseq2 (Galaxy Version 2.11.40.6 + galaxy1) [53]. Here, four pairwise comparisons were performed: AV vs. AM; HV vs. HM; HM vs. AM; and HV vs. AV. Total read counts for each miRNA were normalized by median ratio normalization. The resulting *p*-values were corrected for multiple testing using Benjamini and Hochberg’s false discovery rate (FDR) [54]. MiRNAs were judged to be DE if the FDR-adjusted *p*-value (Padj) was ≤0.01 and log2 fold-change was >1.0 or <−1.0. Heatmaps were created using bioinfokit python package (Version 2.0.8) to visualize significantly DE miRNAs [55].

### 2.6. miRNA Target Prediction and Enrichment Analysis

PsRNAtarget (2017 release, https://www.zhaolab.org/psRNATarget/home, accessed on 2 February 2021), with a strict expectation score ≤ 3, was used to predict miRNA targets [56]. Mature miRNA sequences were aligned against transcript sequences retrieved from the Plaza 4.0 dicot database. Subsequently, the Plaza 4.0 workbench was used to obtain gene ontology (GO) terms according to the molecular function, biological process, and cellular component with default parameters [57].

Single enrichment analysis (SEA) was performed using agriGO v2.0 (http://systemsbiology.cau.edu.cn/agriGOv2/, accessed on 3 February 2021) [58]. The GO terms of targets predicted from all identified miRNAs were input as a custom background list. Since separate analyses of up- and downregulated genes could identify pathways that are more relevant to phenotypic differences, the GO terms of those targets predicted from up- or downregulated miRNAs were input separately as a query list [59]. For SEA, Fisher’s exact test with a minimum of 5 mapping entries per term was selected as the statistical test, and the Benjamini–Yekutieli FDR with a significance level of 0.05 was selected as the correction method [60].

### 2.7. Evaluation of the Expression of miRNAs and Their Potential Target Genes

The abundance of selected miRNAs was examined using linear specific (S)-poly (A)-tailed real-time RT-PCR, as described by Xie and collaborators [61,62,63]. Total RNA purification and DNase treatment were performed using the methods described in Section 2.2 above. DNase-treated RNAs were then polyadenylated using poly (A) polymerase (New England BioLabs, Ipswich, MA, USA) according to the manufacturer’s instructions. For each miRNA, cDNA was synthesized from polyadenylated RNA (1 µg) by using Superscript IV reverse transcriptase (Invitrogen, Carlsbad, CA, USA) with S-poly (A)-tailed RT primers (Table S1). These RT primers (SRT) that target specific miRNAs were designed using sRNAprimerDB software version 1.0 (http://www.srnaprimerdb.com/, accessed in May 2021) [63]. After cDNA synthesis, real-time PCR was performed using a SensiFAST SYBR No-ROX kit (Bioline, London, UK) following the manufacturer’s instructions with cycling conditions of 95 °C for 2 min, followed by 40 cycles of 95 °C for 5 s, 61 °C for 10 s, and 72 °C for 10 s. The miRNA-specific primers (SqPF) and universal reverse primer (universal_SqPR) were used for real-time PCR quantification of mature miRNAs (Table S1). For target gene quantification, cDNA was synthesized from DNase-treated RNA using the SensiFAST™ cDNA Synthesis Kit (Bioline, London, UK) following the manufacturer’s instructions with reaction conditions of 25 °C for 10 min, 42 °C for 15 min, 85 °C for 5 min. Then, real-time PCR was performed using the method described above. Based on the transcript sequences obtained from our previous study [13], primers were designed using Geneious Prime and are listed in Table S1. U6 and Actin genes were used as internal controls for miRNAs and target genes quantification, respectively [64,65] (Table S1). Relative expression levels of mature miRNAs and target genes were calculated by the 2^−ΔΔCT^ method [66].

Northern blot hybridization was used for the detection and quantification of mature Can-miR164b/c since a specific product of mature miRNA164b/c was unable to be amplified by using linear S-poly (A)-tailed real-time RT-PCR. Total RNA was separated on a 17% polyacrylamide gel [67]. Subsequentially, gel-separated RNA was transferred onto a Hybond-N+ nylon membrane (Roche) using a Bio-Rad mini trans-blot system. For U6 and Can-miR164b/c detection, DIG-labeled DNA probes of U6 (5′-TCATCCTTGCGCAGGGGCCA) and Can-miR164b/c (5′-TGCACGTGCCCTGCTTCTCCA) were generated using the DIG Oligonucleotide 3′-End Labelling Kit (Roche, Basel, Switzerland). The relative accumulation level of Can-miR164b/c was then calculated by densitometry of the Northern blot chemiluminescent images using iBright analysis software (Version 5.0.0). The densitometry value of the AM condition was set as reference in each blot. The normalized U6 values served as an internal control for the normalized values of Can-miR164b/c.

Statistical tests were performed using GraphPadPrism software (Version 9.3.1). Data were analyzed with unpaired Student’s *t*-tests and considered significantly different if the two-tailed *p*-value was <0.05.

### 2.8. Cleavage Sites Validation of Target Genes of miRNAs

Modified 5′ RNA ligase-mediated amplification of cDNA ends (5′-RLM-RACE) was used to validate the cleavage sites of predicted miRNA target genes [68,69]. This modified procedure starts with ligating the 5′-RNA adapter (RLM_RNA_adapter, Table S1) to total RNAs using T4 RNA ligase (New England Biolabs, Ipswich, MA, USA). Then, the adapter-ligated RNAs were used to synthesize first-strand cDNA using Superscript IV reverse transcriptase (Invitrogen) with the oligo dT primer following the manufacturer’s instructions. The cDNA was used as the template for touchdown PCR using Phusion High-Fidelity DNA Polymerase (New England Biolabs, Ipswich, MA, USA) with the primers RLM, gene-specific primers (RLM_GSP)—including RLM_AGO1b_GSP, RLM_LAC4_GSP, and RLM_CLAVATA_GSP (Table S1)—and GeneRacer_5P. The cycling program of touchdown PCR was set as the initial annealing temperature of 68 °C with 3 cycles, followed by a reduced annealing temperature of 66 °C with 3 cycles, 63 °C with 7 cycles, and 60 °C with 22 cycles. The products of the first amplification were used as the templates for the second PCR with nested primer pairs RLM_nGSP/GeneRacer_n5P (Table S1) that annealed internally to the first primer pairs. The products of the second PCR were then cloned into pGEM^®^-T Easy vector (Promega, Madison, WI, USA) and sequenced to analyze the cleavage sites.

## 3. Results

### 3.1. CaCV Symptom Development in Resistant Capsicum Plants at Elevated Temperatures

**Figure 1:**
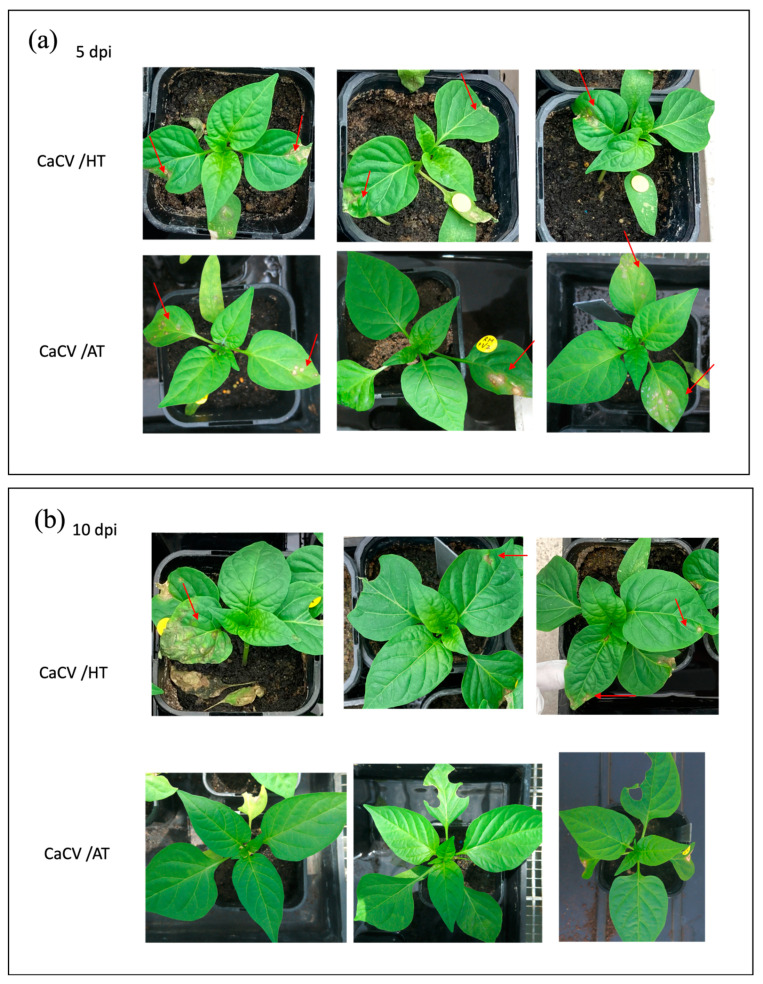
Effect of elevated temperature on capsicum chlorosis virus (CaCV) infection in CaCV-resistant capsicum plants: (**a**) at 5 days post inoculation (dpi), a hypersensitive response (red arrows) was apparent in CaCV-inoculated leaves of capsicum plants grown at high temperature (HT) of 35 °C and ambient temperature (AT) of 25 °C; (**b**) at 10 dpi, necrotic spots (indicated by red arrows) were apparent on systemic leaves of some capsicum plants grown at HT but not on any plants grown at AT. Three representative plants are shown for each treatment.

To evaluate the effect of elevated temperature on capsicum resistance to CaCV, symptoms were observed over a 10-day period in CaCV-infected capsicum plants grown at HT and AT. Four-week-old CaCV-resistant capsicum plants grown at AT were inoculated with CaCV prior to half of them being transferred to HT. At 5 dpi, all resistant plants challenged with CaCV showed HR-mediated necrotic spots on virus-inoculated leaves at both AT and HT (Figure 1a). At 10 dpi, 8 out of 14 (57%) CaCV-infected plants grown at HT showed distinct necrotic spots on upper systemic leaves, while none of the CaCV-infected plants grown at AT had symptoms on the systemic leaves (Figure 1b). All mock-inoculated CaCV-resistant plants grown at HT or AT remained symptomless and had a similar appearance at the two temperatures. These results indicate that the HR was successfully triggered by CaCV infection in the inoculated leaves of CaCV-resistant capsicum, which was independent of growth temperature. However, the HR appears to be unable to restrict CaCV infection in some plants grown at HT, allowing CaCV to move to systemic leaves and trigger HR there.

### 3.2. Small RNAs in CaCV-Infected and Virus-Free Capsicum Grown at Different Temperatures

**Figure 2:**
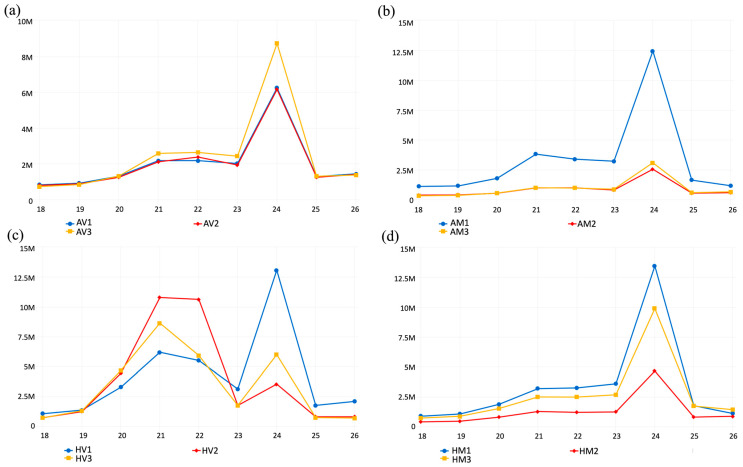
Small RNA length distribution of 18-nucleotide to 26-nucleotide (million (M) reads) in 12 small RNA libraries. Each diagram represents three independent libraries constructed from samples collected from (**a**) capsicum chlorosis virus (CaCV)-inoculated plants grown at ambient temperature (AV), (**b**) mock-inoculated plants grown at ambient temperature (AM), (**c**) CaCV-inoculated plants grown at higher temperature (HV), and (**d**) mock-inoculated plants grown at higher temperature (HM).

**Figure 3:**
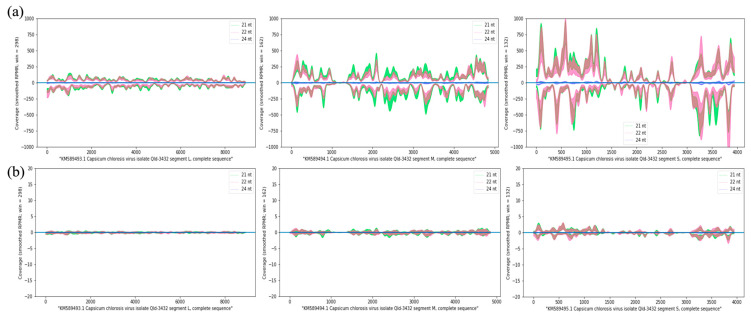
Profiles of capsicum chlorosis virus (CaCV)-derived viral siRNAs. The 21-, 22-, and 24-nucleotide vsiRNA coverage across the CaCV genome L, M, and S segments in CaCV-infected capsicum plants grown at (**a**) higher temperature (HV), and (**b**) ambient temperature (AV). Standard error of 3 biological replicates is presented as the smoothed plots. The reads-per-million (RPM) scale is set at ±1000 for three datasets of the HV treatment and at ±20 for three datasets of the AV treatment.

To identify miRNAs that may be involved in capsicum resistance to CaCV at HT and/or AT, systemic leaves from three CaCV-infected and three mock-inoculated plants grown at either temperature were collected. For HV treatment, leaf disks were sampled from three plants with systemic necrotic spot symptoms. A total of twelve sRNA libraries, containing three libraries in each of the four conditions, were generated for Illumina sequencing and the data are summarized in Table S2. In total, 123,888,579, 95,340,112, 160,227,368, and 127,753,253 raw reads were obtained from the libraries of the AV, AM, HV, and HM treatments, respectively. After trimming adaptor sequences and low-quality reads, 121,643,325, 93,441,595, 157,681,773, and 125,749,412 cleaned reads, and 58,305,917, 45,565,384, 102,316,388, and 65,410,603 length-limited reads remained for AV, AM, HV, and HM treatments, respectively (Table S2). In the analysis of length distribution, 24-nucleotide sRNAs were the largest sRNA group among all length-limited reads in the libraries of AV, AM, and HM treatments (Figure 2a,b,d), whereas 21-nucleotide and 22-nucleotide sRNAs accounted for the highest percentage of all sRNAs in the HV libraries in addition to the 24-nucleotide sRNA peak (Figure 2c). To further analyze vsiRNAs and miRNAs, length-limited reads were aligned to the capsicum genome. An average of 70.0%, 70.2%, 56.4%, and 73.3% of reads for the AV, AM, HV, and HM treatments, respectively, mapped to the capsicum reference genome. The reads that failed to map to the capsicum genome in the AV and HV libraries were retrieved for vsiRNA analysis and indicated that the expression of CaCV-derived vsiRNAs was significantly higher in CaCV-infected capsicum grown at HT than in those plants grown at AT (Figure 3, compare panels a and b). Visualization of vsiRNA coverage in CaCV-infected capsicum grown at HT showed that vsiRNAs were more evenly dispersed across the entire L segment than across M and S segments (Figure 3a). Viral siRNAs were less abundant across the intergenic region of both M and S segments, indicating vsiRNA hotspots in the coding regions of M and S segments (Figure 3a). Moreover, the abundance of 21-nucleotide, 22-nucleotide, and 24-nucleotide vsiRNAs at the higher temperature was highest in several hotspots in the CaCV S segment in all three HV libraries (Figure 3a).

### 3.3. Identification of Known and Novel miRNAs

The sRNA datasets were grouped according to the four treatments (AV, AM, HV, and HM), and the tool miRDeep-P2 in iwa-miRNA software was used to predict miRNA clusters. The capsicum miRNAs were annotated using the miRNA annotations retrieved from PmiREN, sRNAanno, and PsRNA databases. After aggregating annotated and predicted miRNAs into a candidate miRNAs list, miRNAs that passed both high-throughput-based and machine learning-based criteria were selected. From the four grouped datasets, 105 known and 83 novel miRNAs were identified. Among all identified miRNAs, 89 known and 47 novel miRNAs were present in libraries across more than one treatment. In addition, 69 known and 21 novel miRNAs were identified in all libraries (Figure S1). The mature, star, and precursor sequences of identified miRNAs present in libraries across more than one treatment are listed in Table S3. The abundance of miRNAs in individual sRNA libraries was determined by aligning reads against the mature, star, and precursor sequences of those identified miRNAs. They were given the prefix ‘Can-’ for *Capsicum annuum*. Overall, Can-MIR159 and Can-MIR166 were the most abundant known miRNA families and Can-MIRN19 was the most abundant novel miRNA family in all libraries across all treatments.

### 3.4. Differentially Expressed miRNAs

**Figure 4:**
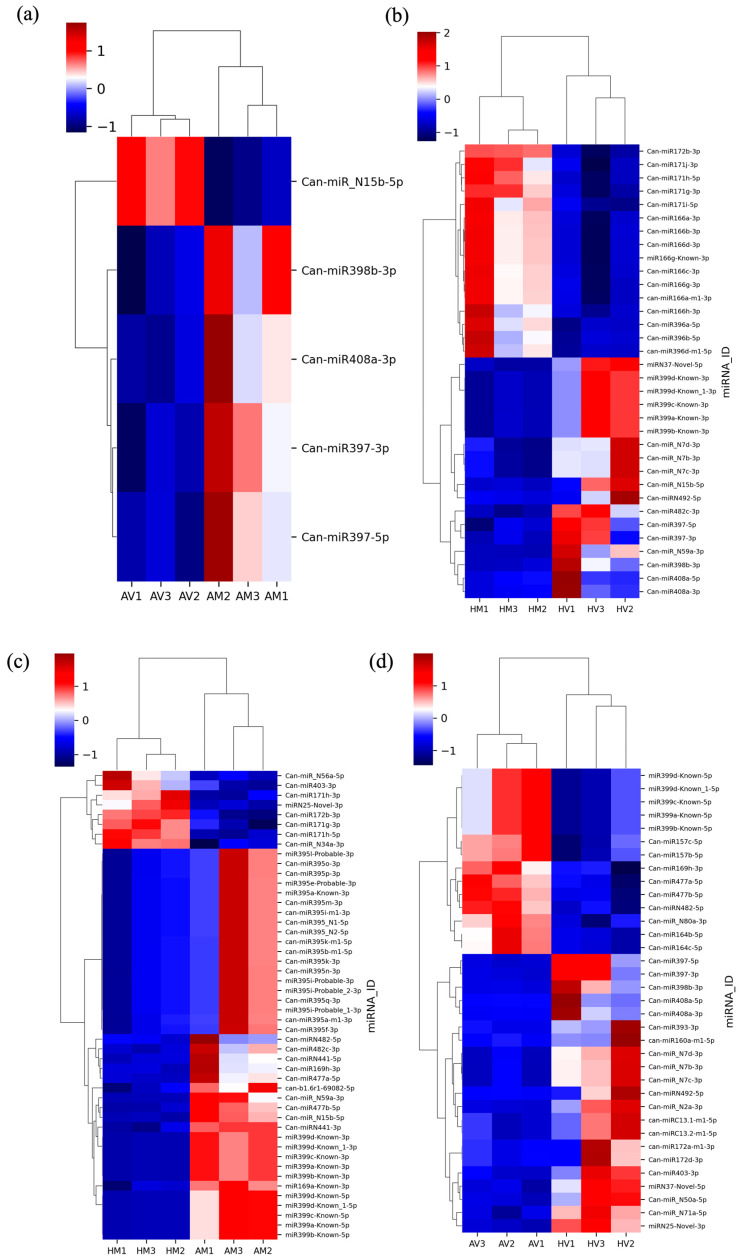
Heat maps showing differentially expressed capsicum miRNAs in four pairwise comparisons: (**a**) capsicum chlorosis virus (CaCV)-infected capsicum grown at ambient temperature (AT) compared to mock-inoculated capsicum grown at AT (AV vs. AM); (**b**) CaCV-infected capsicum grown at higher temperature (HT) compared to mock-inoculated capsicum grown at HT (HV vs. HM); (**c**) mock-inoculated capsicum grown at HT compared to AT (HM vs. AM); (**d**) CaCV-infected capsicum grown at HT compared to AT (HV vs. AV); Columns represent independent biological replicates; rows represent different miRNAs. Clustering is based on Z-score hierarchical calculation. Red and blue indicate miRNAs with high and low expression, respectively, as shown on the color scale.

The differential expression of miRNAs was calculated using DEseq2 in four pairwise comparisons: AV vs. AM; HV vs. HM; HM vs. AM; and HV vs. AV. Five DE miRNAs were identified at AT when comparing CaCV-infected and mock-inoculated treatments, which included one upregulated and four downregulated miRNAs (Figure 4a). In HV vs. HM and HM vs. AM comparisons, 34 and 48 DE miRNAs, respectively, were detected. Of those DE miRNAs, 18 upregulated and 16 downregulated miRNAs were identified in the HT CaCV-infected treatment versus mock treatment; 8 upregulated and 40 downregulated miRNAs were identified in mock treatments comparing HT and AT conditions (Figure 4b,c).

To identify miRNAs that are potentially involved in temperature-sensitive resistance to CaCV, we focused on the comparative expression levels of miRNAs in HV and AV conditions. A total of 35 DE miRNAs, including 21 upregulated and 14 downregulated miRNAs, were identified at HT compared to AT in CaCV-infected plants (Figure 4d). Among those, DE miRNAs, Can-MIR408, Can-MIR398, Can-MIR397, Can-MIR393, Can-MIRN492, and Can-MIRN7 were the most highly upregulated miRNA families with log2-fold changes above 2.0 in HV compared to AV conditions (Table 1). Interestingly, Can-miR408a-3p, Can-miR398b-3p, Can-miR397-3p, and Can-miR397-5p were upregulated when comparing the HV and HM treatments but were downregulated when comparing the AV and AM treatments (Table 1). For those downregulated miRNAs in the CaCV-infected treatment comparing HT and AT growth conditions, some miRNAs in the Can-MIR169, Can-MIR477, Can-MIRN482, and Can-MIR399 families were responsive to HT and showed a reduced expression in HM versus AM treatment. Notably, Can-miR157b-5p, Can-miR157c-5p, Can-miR164b-5p, Can-miR164c-5p, and Can-miR_N80a-3p were downregulated only in HV compared to AV treatment but not in HM compared to AM treatment (Table 1). These data indicate that increased or reduced expression of some miRNAs in Can-MIR408, Can-MIR398, Can-MIR397, Can-MIR157, Can-MIR164, and Can-MIR_N80a families may be associated with CaCV-induced changes between HT and AT treatments.

### 3.5. Functional Analysis of Predicted Target Genes of DE miRNAs in Response to CaCV under Ambient and High-Temperature Conditions

For a better understanding of the potential regulatory roles of DE miRNAs during CaCV infection and at elevated temperature, target genes of all identified miRNAs were predicted by psRNAtarget software with stringent criteria (expectation score ≤ 3) and were functionally annotated with GO terms. An overview of all DE miRNAs and their predicted mRNA targets in the four pairwise comparisons AV vs. AM, HV vs. HM, HM vs. AM, and HV vs. AV is shown in the Venn diagrams depicted in Figure S2. A large proportion of the upregulated miRNAs and their predicted targets in HV compared to AV overlapped with those upregulated miRNAs and their predicted targets in HV compared to HM treatments (Figure S2a,c), suggesting a significant virus effect. However, downregulated miRNAs and their predicted targets in HV compared to AV overlapped with those downregulated miRNAs and their predicted targets in HM compared to AM (Figure S2b,d), suggesting a significant temperature effect.

**Figure 5:**
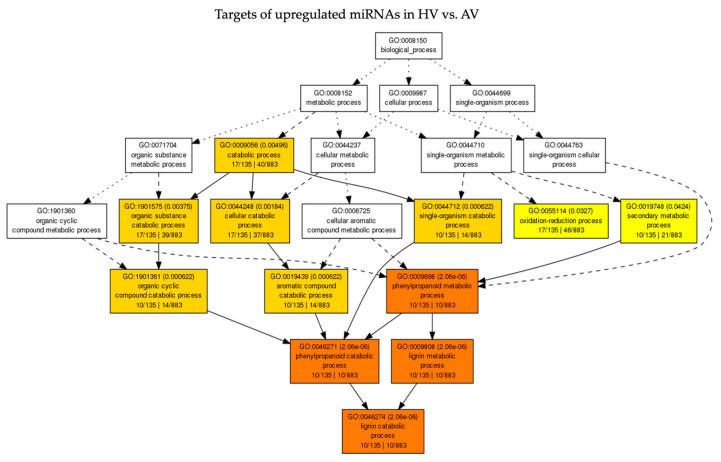
Acyclic graph showing hierarchical relationship of the enriched gene ontology (GO) terms associated with targets predicted from upregulated miRNAs in CaCV-infected capsicum grown at high temperature (HV) compared to ambient temperature (AV) in the biological process category. The color scale from yellow to red indicates an increasingly significant enrichment of GO terms.

**Figure 6:**
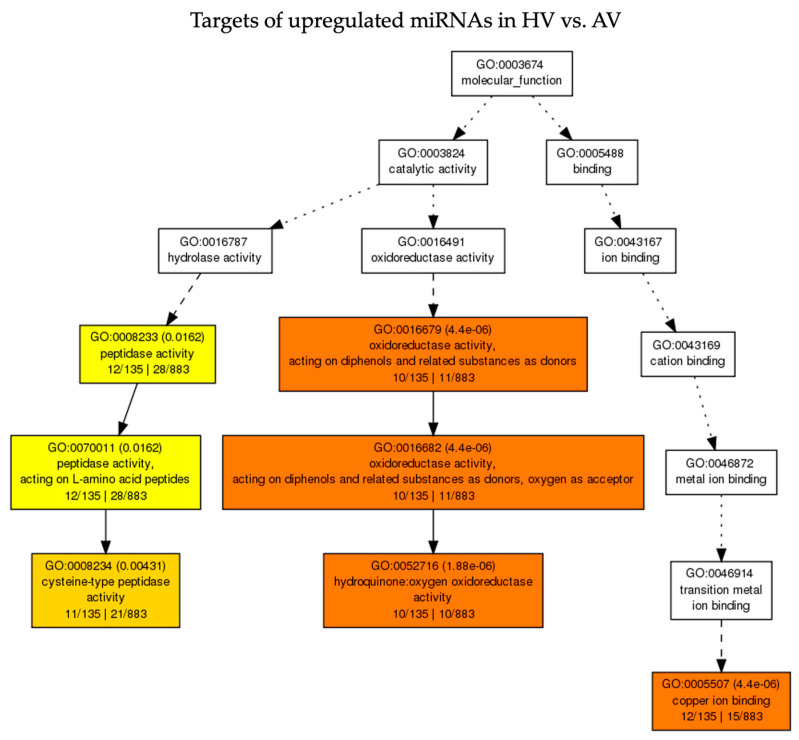
Acyclic graph showing hierarchical relationship of the enriched gene ontology (GO) terms associated with targets predicted from upregulated miRNAs in CaCV-infected capsicum grown at high temperature (HV) compared to ambient temperature (AV) in the molecular function category. The color scale from yellow to red indicates an increasingly significant enrichment of GO terms.

To identify pathways that are relevant to the observed phenotypic differences between HV and AV treatments, GO enrichment analyses were conducted for the targets that were predicted from up- or downregulated miRNAs. Besides analyzing targets of DE miRNAs in HV vs. AV treatments, targets of DE miRNAs in other pairwise comparisons (AV vs. AM, HV vs. HM, and HM vs. AM) were also included. All significantly enriched (Padj  <  0.05) GO terms describing biological process (BP), molecular function (MF), and cellular component (CC) for the predicted targets of DE miRNAs are listed in Tables S4–S9. A total of 156 targets were predicted from 21 miRNAs that were upregulated in CaCV-infected plants at HT compared to AT. Among them, 143 miRNA targets could be functionally annotated. Subsequently, 8 and 12 GO terms listed in MF and BP were enriched through the SEA of those miRNA targets. The hierarchical relationships of the significantly enriched terms for BP and MF are presented in Figure 5 and Figure 6, respectively. DE miRNAs and their corresponding target genes with enriched GO terms are shown in Table S4. Briefly, miRNA targets that were annotated with significantly enriched GO terms in BP and MF categories—including the phenylpropanoid metabolic process (GO:0009698), lignin catabolic process (GO:0046274), lignin metabolic process (GO:0009808), phenylpropanoid catabolic process (GO:0046271), hydroquinone: oxygen oxidoreductase activity (GO:0052716), and copper ion binding (GO:0005507)—were regulated by Can-miR408a-3p and Can-miR397-5p (Figure 5 and Figure 6 and Table S4). In agreement with the relevance of DE miRNAs among the pairwise comparisons HV vs. AV, HV vs. HM, and AV vs. AM, the most highly enriched GO terms were also enriched through SEA of targets of downregulated miRNAs in AV compared to AM as well as targets of upregulated miRNAs in HV compared to HM (Figures S3 and S4). In addition, the GO-enriched term, cysteine-type peptidase activity (GO:0008234) for MF, was associated with a group of targets that were affected by upregulated miRNAs (Can-miR_N7b-3p, Can-miR_N7c-3p, Can-miR_N7d-3p, miRN37-Novel-5p, and Can-miR408a-5p) in HV compared to both AV and HM treatments (Figure 6, Figure S4 and Tables S4 and S6).

For miRNAs that were downregulated at higher temperature in CaCV-infected plants, a total of 48 target mRNAs were functionally annotated. Among those targets, only two GO terms (GO:0003677 and GO:0003676) were enriched in the MF category. Moreover, these two enriched terms were associated with targets that were predicted from eight miRNAs, including Can-miR157b-5p, Can-miR157c-5p, Can-miR164b-5p, Can-miR164c-5p, Can-miR169h-3p, Can-miR477a-5p, Can-miR_N80a-3p, and Can-miRN482-5p (Table S5). Based on the functional prediction of miRNA targets and the DE miRNAs, Can-miR408a-3p, Can-miR397-5p, Can-MIR157, and Can-MIR164 families may represent important known miRNAs involved in temperature-dependent resistance-breaking.

### 3.6. Validation of the Expression Patterns of Selected miRNAs and Their Target Genes

**Figure 7:**
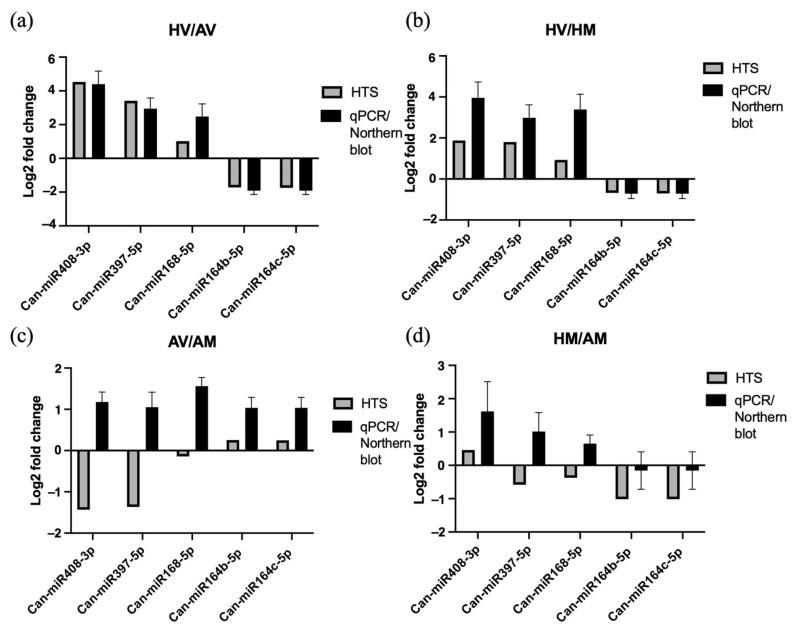
Linear specific (S)-poly (A)-tailed quantitative real-time RT-PCR (qPCR) of miR408, miR397, and miR168 or Northern blot hybridization confirmation of miR164b and miR164c expression patterns compared to those obtained by sRNA high-throughput sequencing (HTS). The expression levels (log2-fold change) of five miRNAs in four pairwise comparisons: (**a**) CaCV-infected plants at high temperature (HV) vs. CaCV-infected plants at ambient temperature (AV); (**b**) HV vs. mock-inoculated plants at high temperature (HM); (**c**) AV vs. mock-inoculated plants at ambient temperature (AM); and (**d**) HM vs. AM are displayed for HTS and qPCR or Northern blot. *U6 * was used as internal reference for calibrating the expression of miRNAs. The error bars represent the mean (±standard error of the mean) of 4 biological replicates.

To confirm the sRNA sequencing data, S- poly(A)-tailed real-time RT-PCR or Northern blot with signal intensity quantification was used to quantify the levels of selected miRNAs at 10 dpi. Based on the results of the differential expression analysis of miRNAs and functional analysis of targets, Can-miR408a-3p, Can-miR397-5p, and Can-miR164b/c-5p were selected based on their potential involvement in temperature-dependent resistance-breaking. In addition, Can-miR168-5p, which was upregulated with a log2-fold change of 1.0 and Padj of 0.017 in HV compared to the AV condition, was selected despite the cut-off threshold of Padj ≤ 0.01. This exception was based on the reported importance of miR168. Accumulation of miR168 compromises AGO1-mediated antiviral RNA silencing in several virus-infected plant species [70,71,72]. In addition, accumulation of miR168 may affect the expression of many target genes since AGO1 plays a central role in RNA silencing [73]. The results of the real-time RT-PCR and Northern blot (Figure S5) confirmed the increased expression levels of Can-miR408a-3p, Can-miR397-5p, and Can-miR168-5p as well as the decreased expression level of Can-miR164b/c-5p in HV compared to AV treatments, and showed that they were consistent with the expression profiles obtained by sRNA sequencing (Figure 7a). When HV was compared to HM, the expression trends of Can-miR408a-3p, Can-miR397-5p, and Can-miR164b/c-5p were similar between the results observed in real-time RT-PCR and sRNA sequencing (Figure 7b). However, expression trends of Can-miR408a-3p, Can-miR397-5p, and Can-miR168-5p in the AV and AM comparison were opposite between the results of real-time RT-PCR and sRNA sequencing (Figure 7c).

**Figure 8:**
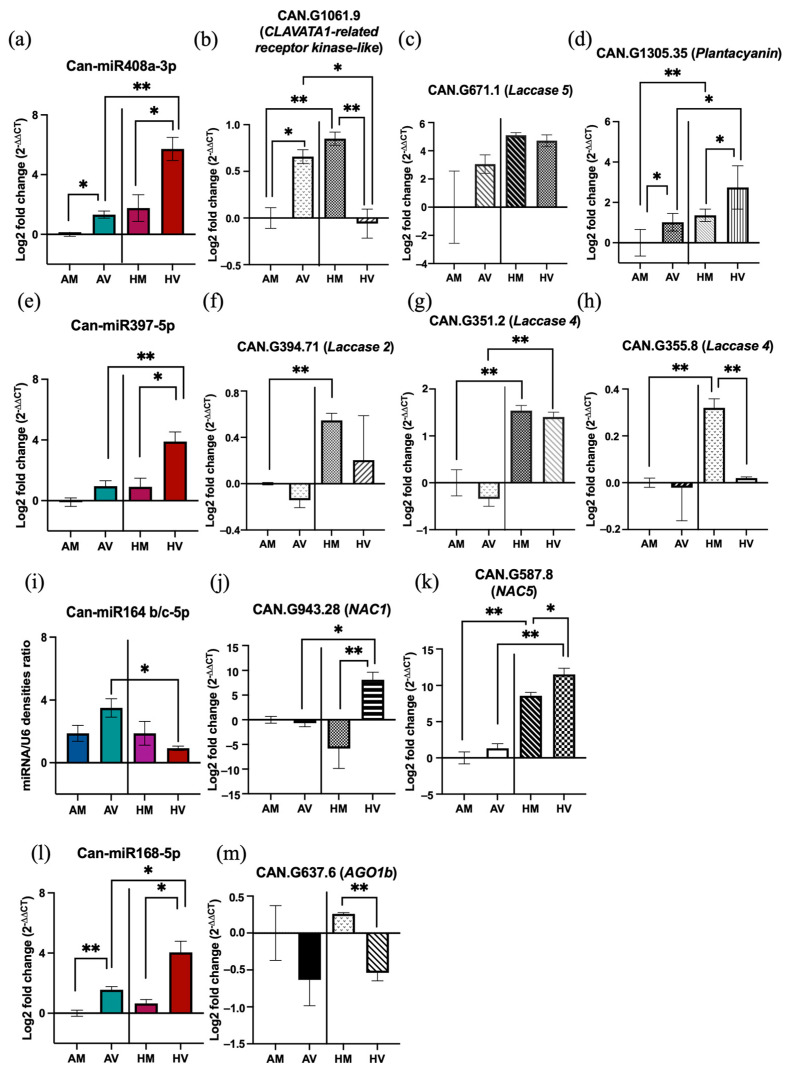
MicroRNA-mediated regulation involved in capsicum resistance response to CaCV at high temperature (HV) or ambient temperature (AV). Expression patterns of miRNAs (in color) and targets (in black and white) in four pairwise comparisons (AV vs. mock-inoculated plants at ambient temperature (AM); HV vs. mock-inoculated plants at high temperature (HM); HM vs. AM; and HV vs. AV) were analyzed by real-time RT-PCR with the log2-fold change 2–ΔΔCt method or by Northern blot. Real-time RT-PCR of (**a**) Can-miR408a-3p and its targets: (**b**) CAN.G1061.9, (**c**) CAN.G671.1, and (**d**) CAN.G1305.35. Real-time RT-PCR analysis of (**e**) Can-miR397-5p and its targets: (**f**) CAN.G394.71, (**g**) CAN.G351.2, and (**h**) CAN.G355.8. Northern blot analysis of (**i**) Can- miR164b/c-5p, and real-time PCR analysis of its targets: (**j**) CAN.G394.28 and (**k**) CAN.G587.8. Real-time RT-PCR analysis of (**l**) Can-miR168-5p and its target (**m**) CAN.G637.6. Northern blot analysis was quantified through measuring signal strength using ibright. Actin and U6 were used as internal reference for calibrating the expression of targets and miRNAs, respectively. The error bars represent the mean (±standard error of the mean) of 4 biological replicates. Significant differences between treatments were assessed with Student’s *t*-test (* *p* < 0.05; ** *p* < 0.01).

In addition to analyzing the expression of mature miRNAs, quantitative real-time RT-PCR was also used to investigate the effect of these miRNAs on their target genes in the HV condition. Targets were selected based on the target prediction data obtained from psRNAtarget and WPMIAS software [74] as well as the target validation data reported by Zhang and collaborators [75]. CAN.G1061.9 (*CLAVATA1-related*), CAN.G671.1 (*Laccase 5*, *LAC5*), and CAN.G1305.35 (*Plantacyanin*) were chosen for Can-miR408a-3p target analysis; CAN.G394.71 (*LAC2*), CAN.G351.2 (*LAC4*), and CAN.G355.8 (*LAC4*) were chosen for Can-miR397-5p target analysis; CAN.G637.6 (*AGO1b*) was chosen for Can-miR168-5p target analysis; NAM/ATAF1,2/CUC2 (*NAC*) transcription factors—CAN.G943.28 (*NAC1*) and CAN.G587.8 (*NAC5*)—were chosen for Can-miR164b/c-5p target analysis. Unexpectedly, expression patterns of the targets CAN.G671.1, CAN.G1305.35, CAN.G394.71, CAN.G351.2, CAN.G355.8, and CAN.G637.6 were not inverse to the expression of their corresponding miRNAs when comparing the HV and AV conditions (Figure 8c,d,f–h,m). Conversely, the expected inverse expression pattern was observed in Can-miR408a-3p/CAN.G1061.9, Can-miR164b/c-5p/CAN.G943.28, and Can-miR164b/c-5p/CAN.G587.8 pairs (Figure 8b,j,k). Notably, we found that the transcript levels of all three selected targets of Can-miR397-5p in mock-inoculated plants drastically increased at HT (Figure 8f–h). Moreover, the transcript level of one of the targets, CAN.G355.8, was downregulated, while that of the other two targets (Figure 8h), CAN.G394.71 and CAN.G351.2, remained unchanged in CaCV-infected plants as compared with mock-inoculated plants at HT (Figure 8f,g). These results suggest that the downregulation of CAN.G355.8 may be due to the negative regulation of upregulated Can-miR397-5p in HV compared to HM. Similarly, an inverse expression pattern between Can-miR168-5p and CAN.G637.6 was also observed in HV vs. HM (Figure 8m). Different from other targets that showed inverse or no correlation tendency with miRNAs, CAN.G1305.35 showed a positively correlated expression pattern with Can-miR408a-3p in three pairwise comparisons (AV vs. AM, HV vs. HM, and HV vs. AV) (Figure 8d). Overall, diverse regulatory effects of miRNAs on their targets were observed when plants were treated at different temperatures and biotic stress conditions.

### 3.7. Identification of miRNA-Mediated Cleavage of Target Genes

**Figure 9:**
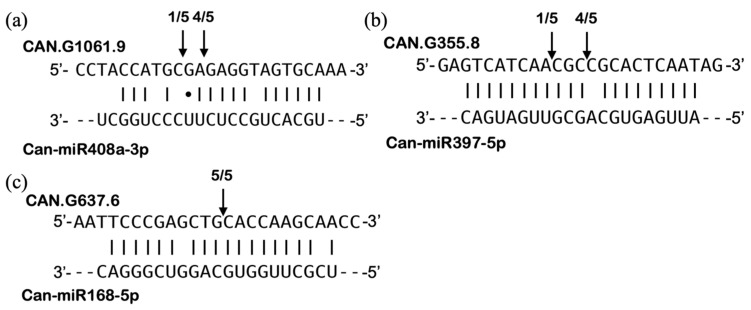
Validation of miRNA targets by 5′ RLM-RACE. The potential cleavage sites of (**a**) Can-miR408a-3p on CAN.G1061.9, (**b**) Can-miR397-5p on CAN.G355.8, (**c**) Can-miR168-5p on CAN.G637.6 in capsicums were mapped. The arrows indicate the cleavage sites and the numbers indicate clone frequencies. The dashes represent the standard base pairing rules and the dots indicate GU wobble base pairing.

To confirm putative miRNA targets, the specific cleavage sites were identified experimentally using 5′-RACE. Three targets, including CAN.G1061.9, CAN.G355.8, and CAN.G637.6, were chosen for the 5′-RACE verification based on the observed inverse expression patterns of miRNA/target pairs. CAN.G1061.9 was selected for its inverse expression pattern at HV vs. AV, while CAN.G355.8 and CAN.G637.6 were chosen for their inverse patterns at HV vs. HM. The other targets, CAN.G943.28 and CAN.G587.8, which showed opposite correlation with Can-miR164b/c-5p, were verified by degradome-based miRNAs-target analysis using WPMIAS (Table S10). Based on the results of 5′-RACE, the cleavage sites were all located in the regions that were predicted as miRNA-binding sites (Figure 9). The majority cleavage site in CAN.G1061.9 was mapped to the 11th position of the complementary sequence at the 5′-end of Can-miR408a-3p (Figure 9a). On the other hand, the main cleavage site in CAN.G355.8 and CAN.G637.6 was mapped to the 10th position of the complementary sequences at the 5′-end of Can-miR397-5p and Can-miR168-5p, respectively (Figure 9b,c). These results suggest that CAN.G1061.9, CAN.G355.8, and CAN.G637.6 can be cleaved by Can-miR408a-3p, Can-miR397-5p, and Can-miR168-5p, respectively, in capsicum.

## 4. Discussion

The suppressive effect of elevated temperatures on ETI-mediated resistance or its associated HR response has been demonstrated in both non-viral [20,76,77] and viral pathosystems [20,78]. For example, the *N* gene-mediated HR and resistance to TMV in tobacco are compromised by exposing plants to temperatures above 28 °C [20,78]. In the present study, we found that ETI-mediated resistance to CaCV was overcome at 35 °C/30 °C (day/night) in 8 out of 14 capsicum plants. Interestingly, instead of abolishing HR, distinct local lesions developed in inoculated leaves, and systemic necrotic lesions were also observed in these plants in which resistance is compromised at 35 °C. This result agrees with the previous finding suggesting that HR may fail to restrict virus movement in local lesions at elevated temperatures [26,27,79]. At 32 °C, the *Tsw*-mediated resistance to TSWV in capsicum is compromised, leading to virus movement into uninoculated leaves. Nonetheless, local HR persists in the inoculated leaves of those plants [26,27,79]. Notably, there is limited knowledge regarding the mechanisms that underpin temperature-sensitive ETI-mediated resistance in viral pathosystems [20,29]. In the TMV–tobacco pathosystem, HR is suppressed at elevated temperatures by preventing NLR proteins from translocating into the nucleus to orchestrate plant immune signaling. Tobacco plants mount an effective HR response when the host N protein accumulates in the nucleus after recognition of the TMV coat protein at 22 °C, while plants are unable to induce an HR response at 28 °C due to decreased N protein nuclear localization [20]. In addition, changes in miRNA accumulation that affect the expression of their target genes appear to be linked to the temperature sensitivity of Ny-DG-mediated resistance in the PVY–potato pathosystem. Resistance to PVY^NTN^ was compromised at 28 °C and an enhanced miRNA-mediated downregulation of a specific NLR transcript was observed in PVY-inoculated potato leaves [29].

In previous studies, several miRNAs have been identified in *Capsicum* spp. [41,80,81,82,83,84]. Some of these miRNAs were potentially involved in the interactions between capsicum and plant pathogens [41,84]. In the present study, Illumina sequencing of sRNAs was conducted to investigate the involvement of miRNAs in temperature-sensitive antiviral resistance. By predicting and retrieving miRNA annotations from three sRNA databases—PmiREN, sRNAanno, and PsRNA—105 known and 83 novel miRNAs were identified across four treatment groups: AV, AM, HV, and HM. Given the distinct phenotypic differences observed between CaCV-infected capsicum plants grown at HT and AT, the DE miRNAs revealed by comparing HV and AV treatments allowed us to identify miRNAs potentially involved in temperature-sensitive resistance. Among the DE miRNAs, a significant number expressed higher in the HV than AV were responsive to CaCV infection at HT. In addition, in the enrichment analysis of the upregulated miRNAs in HV compared to AV, targets of Can-miR408a-3p and Can-miR397-5p—which are associated with biological processes, including the metabolism and catabolism of phenylpropanoids and lignin—were identified as the most enriched GO terms.

Lignin deposition plays a crucial role in plant resistance to biotrophic pathogens by confining them to the infection site [85]. In *A. thaliana*, lignification is induced during the ETI response, triggered by infection of avirulent bacterial strains, *Pseudomonas syringae* pv. tomato (*Pst*) DC3000 (AvrRpm1) and *Pst* DC3000 (AvrRpt2). This lignification is associated with localized cell death, restricting the bacterial pathogens to the infection site [85]. Increased lignin accumulation has also been associated with plant resistance to viral pathogens, including TMV, tobacco necrosis virus, southern bean mosaic viruses, and sweetpotato virus disease (SPVD) [86,87]. We, therefore, speculate that the upregulation of Can-miR408a-3p and Can-miR397-5p in HV compared to AV may reduce lignin content by targeting genes in the *LAC* family, which in turn may interfere with restrictions to virus spread from the infection site. Although there is no direct evidence showing the involvement of miR408 and miR397 in lignin-related HR, reduced lignin deposition has been observed in plants overexpressing these two miRNAs (Lu et al. 2013; Song et al. 2018). Moreover, inhibition of miR397 has been shown to increase lignin deposition in the cell wall and reduce the accumulation of SPVD [87].

Based on computational results and their involvement in plant responses to various stresses [88], Can-miR408a-3p, Can-miR397-5p, Can-miR168-5p, and Can-miR164b/c-5p were selected for further analysis of sRNA and target genes expression profiles. Real-time RT-PCR revealed that Can-miR408a-3p, Can-miR397-5p, and Can-miR168-5p were upregulated, whereas Can-miR164b/c-5p was downregulated in HV compared to AV. Additionally, Can-miR408a-3p and Can-miR397-5p were upregulated in HV compared to HM, consistent with our sRNA sequencing data. However, discrepancies were observed between the results of sRNA sequencing and real-time RT-PCR in some pairwise comparisons. For instance, in AV and AM comparisons, real-time RT-PCR indicated an increased level of Can-miR408a-3p and an unchanged level of Can-miR397-5p. Conversely, sRNA sequencing showed decreased levels for both. This inconsistency between sequencing and RT-PCR data has also been documented in prior studies [89,90]. Several factors may contribute to discrepancies between computational sequence analysis and Northern blot or real-time RT-PCR. For instance, biases during sRNA library preparation and amplification, such as adapter ligation bias and varying GC content among miRNAs, can introduce distortions [91,92,93].

Genes that encode copper-containing proteins, including proteins in the phytocyanin family—cupredoxin, plantacyanin, and uclacyanin—or proteins involved in lignin polymerization such as LACs, have been validated as targets of miR408 in *A. thaliana* [94,95]. For miR397, genes in the *LAC* family are its major target genes in various plant species [96,97,98,99,100]. In the present study, GO enrichment analysis suggests that *LAC* family genes, predicted to be regulated by Can-miR408a-3p and Can-miR397-5p, may be crucial for resistance breakdown at HT. Unexpectedly, real-time RT-PCR analysis did not reveal significant differences in the expression levels of the selected *LAC* genes, including CAN.G671.1, CAN.G394.7, CAN.G351.2, and CAN.G355.8, between plants treated with HV and AV. Their expression was not inversely correlated with the increased pattern of Can-miR408a-3p and Can-miR397-5p. However, negative regulation of CAN.G355.8 by Can-miR397-5p was observed in HV compared to HM conditions, wherein the CAN.G355.8 level was reduced, while the Can-miR397-5p level was increased in HV-treated plants. Furthermore, transcript levels of selected Can-miR397-5p targets, including CAN.G394.7, CAN.G351.2, and CAN.G355.8, were increased by HT in mock-inoculated plants. Given that reduced lignin content is detrimental to *Medicago truncatula* growth at HT [101,102], an induction of *LAC*s at HT may be crucial for heat tolerance in capsicum plants. Taken together, we speculate that the suppression of CAN.G355.8 expression by CaCV at HT is likely to reduce plant tolerance to stresses, which may lead to CaCV resistance breakdown at HT.

In addition to the genes in the *LAC* family, the expression of other targets predicted for Can-miR408a-3p was investigated. CAN.G1061.9 (leucine-rich receptor-like kinase *CLV1-related*) was shown to be cleaved by Can-miR408a-3p through 5′-RACE in the present study. The transcript level of CAN.G1061.9 was lower in CaCV-infected plants grown at HT than those grown at AT, which showed an opposite tendency to Can-miR408a-3p in the HV vs. AV comparison. CAN.G1061.9 encodes a CLV1-related receptor kinase-like protein, which appears to be similar to BARELY ANY MERISTEM 1 (BAM2) in the Plaza 4.0 database [57]. Interestingly, BAM2, which functions redundantly with BAM1, participates in the regulation of cell-to-cell RNAi movement [103]. Therefore, a downregulation of CAN.G1061.9 may affect systemic spread of antiviral RNAi, resulting in reduced resistance of capsicum plants to CaCV at HT. Unlike CAN.G1061.9, a negative correlation between miRNA and its target did not occur for Can-miR408a-3p and CAN.G1305.35 (*Plantacyanin*). The transcript level of CAN.G1305.35 increased during Can-miR408a-3p induction in CaCV-infected plants either grown at HT or AT. This agrees with the prior finding that the levels of miR408 and its targets, *Plantacyanin* and *Uclacyanin*, increased simultaneously in late stages of natural senescence in *A. thaliana* [104]. Emerging evidence suggests that a negative correlation between miRNAs and their targets is not strictly required for target validation [105]. Such exceptions may occur when miRNAs and their targets are expressed partially overlapped or in a cell-type-specific manner [105,106,107]. For example, miR395 and its target, *SULTR2;1*, are mainly expressed in phloem companion cells and xylem parenchyma cells in roots, respectively [107]. This, therefore, prevents miR395 from downregulating SULTR2;1, leading to an upregulation of both SULTR2;1 and miR395 in roots during sulfur starvation [107].

In plants, ROS function as central regulators in complex signaling networks [108,109]. The ROS burst can trigger HR and induce plant resistance to viruses [25]. However, excessive ROS accumulation occurs when the homeostasis of ROS is imbalanced, leading to irreversible oxidative damage and accelerated senescence [109]. In our study, miRNA profiles were investigated in systemic, uninoculated leaves that displayed necrotic spots. This necrotic spot phenotype may be associated with an increased accumulation of ROS in systemic leaves at elevated temperature [110]. Interestingly, not only an upregulation of miR408 but also a downregulation of miR164, which have been shown to be associated with accelerated leaf senescence [88,111], were observed in HV compared to AV. Repressing miR164 expression was found to induce the expression of its target gene, *ORE1*, leading to early-senescence phenotypes [111,112]. In addition, *NAC* transcription factors targeted by miR164, such as *ANAC021/22* (*NAC1*), *ANAC079/80* (*NAC4*), and *ANAC100* (*NAC5*), were upregulated during senescence [113]. MiRNA168 is crucial in miRNA and siRNA pathways by targeting AGO1, the core component of the RNA-induced silencing complex [114]. A well-known auto-regulatory feedback loop of miR168 and AGO1 is involved in maintaining their homeostasis [114]. During several viral infections in *A. thaliana* and *N. benthamiana*, increased miR168 levels disrupt this homeostasis, resulting in an inhibition of AGO1-mediated antiviral RNA silencing [71,72]. In our study, the transcript levels of two selected targets, CAN.G943.28 (*NAC1*) and CAN.G587.8 (*NAC5*), were upregulated in HV compared to AV, which was negatively correlated with the downregulated Can-miR164b/c-5p. However, no significant expression differences of CAN.G637.6 (AGO1b) were observed in HV compared to AV. Overall, our findings suggest that the upregulation of Can-miR408a-3p and Can-miR397-5p, as well as the downregulation of Can-miR164b-5p and Can-miR164c-5p, may underpin the temperature-sensitive resistance-breaking phenotype of CaCV-infected capsicum at elevated temperature. The functions of these miRNAs, including regulating lignin deposition and leaf senescence, may affect important cellular mechanisms in CaCV-infected capsicum plants, which may lower plant fitness under stress conditions and further impact the capacity of plant resistance to viruses.

## 5. Conclusions

In this study, we investigated the effect of elevated temperature on a CaCV-resistant capsicum breeding line. Our results showed that ETI-mediated resistance to CaCV is compromised at elevated temperatures (35 °C/30 °C) in some capsicum plants. Through sRNA HTS and computational analysis, we identified 105 known and 83 novel miRNAs across different treatment groups. Of particular interest was the upregulation of Can-miR408a-3p and Can-miR397-5p in HV plants compared to AV among all DE miRNAs. GO enrichment analysis of the target genes (*LAC*s) predicted from these miRNAs suggested the potential involvement of phenylpropanoid (GO:0009698) and lignin (GO:0009808) metabolic processes in the capsicum response to CaCV under different temperatures. MiRNAs, including Can-miR408a-3p, Can-miR397-5p, Can-miR164b/c-5p, and Can-miR168-5p, may underlie the temperature-sensitivity of ETI-mediated resistance in CaCV-infected capsicum plants.

## Figures and Tables

**Table 1:** Differentially expressed miRNAs in virus-infected capsicum versus mock-inoculated capsicum grown at high compared to ambient temperature.

miRNA ID	HV/AV_log2 (FC)	AV/AM_log2 (FC)	HV/HM_log2 (FC)	HM/AM_log2 (FC)
Can-miR408a-5p	4.94651183		2.15675264	
Can-miR408a-3p	4.52786751	−1.431929	1.87576962	
Can-miR398b-3p	4.3647467	−1.1693631	2.7875221	
Can-miR397-5p	3.41052681	−1.3643359	1.80648604	
Can-miR397-3p	3.09698569	−1.1034217	1.41436391	
Can-miRN492-5p	2.4800554		2.07163552	
Can-miR_N7b-3p	2.22044295		1.95841442	
Can-miR_N7c-3p	2.22044295		1.95841442	
Can-miR_N7d-3p	2.16627692		1.84961374	
Can-miR393-3p	2.00053338			
Can-miRN37-Novel-5p	1.97325814		1.2736888	
Can-miR_N2a-3p	1.66675404			
Can-miR172a-m1-3p	1.62135413			
Can-miR172d-3p	1.61286777			
Can-miR_N71a-5p	1.60761055			
Can-miR_N50a-5p	1.51467255			
Can-miR403-3p	1.29105902			1.04987498
Can-miRN25-Novel-3p	1.27226491			1.27136116
Can-miR160a-m1-5p	1.25709852			
Can-miRC13.1-m1-5p	1.19626839			
Can-miRC13.2-m1-5p	1.19626839			
Can-miR157c-5p	−1.0923326			
Can-miR157b-5p	−1.1162378			
Can-miR169h-3p	−1.1385036			−1.8141374
Can-miR_N80a-3p	−1.4595388			
Can-miR477a-5p	−1.6760649			−3.4314472
Can-miR164b-5p	−1.7200209			
Can-miR164c-5p	−1.7417164			
Can-miR477b-5p	−1.7912909			−2.9432463
Can-miRN482-5p	−1.8617146			−2.2237655
Can-miR399a-Known-5p	−1.9889216			−5.5813609
Can-miR399b-Known-5p	−1.9889216			−5.5813609
Can-miR399c-Known-5p	−1.9889216			−5.5813609
Can-miR399d-Known_1-5p	−1.9889216			−5.5813609
Can-miR399d-Known-5p	−1.9889216			−5.5813609

HV/AV_log2 (FC)—log2-fold change when comparing miRNA expression in CaCV-infected capsicum grown at high temperature (HT) with infected plants grown at ambient temperature (AT); AV/AM_log2 (FC)—log2-fold change when comparing miRNA expression in CaCV-infected capsicum grown at AT to that in mock-inoculated capsicum grown at AT; HV/HM_log2 (FC)—log2-fold change when comparing miRNA expression in CaCV-infected capsicum grown at HT to that in mock-inoculated capsicum grown at HT; HM/AM_log2 (FC)—log2-fold change when comparing miRNA expression in mock-inoculated capsicum grown at HT and AT.

## Data Availability

All data can be accessed on the NCBI database under Bioproject PRJNA1128345.

## Supplementary Materials

The following supporting information can be downloaded at: https://www.mdpi.com/article/10.3390/pathogens13090745/s1, Figure S1: Venn diagram of (a) known and (b) novel miRNAs identified in 12 capsicum libraries; Figure S2: Venn diagrams of four pairwise comparisons showing (a) upregulated miRNAs and (c) their corresponding targets, and (b) downregulated miRNAs and (d) their corresponding targets; Figure S3: Acyclic graph showing hierarchical relationship of the enriched gene ontology (GO) terms in the biological process category; Figure S4: Acyclic graph showing hierarchical relationship of the enriched gene ontology (GO) terms in the molecular function category; Table S1: Sequences of oligonucleotide primers used in this study; Table S2: Summary of small RNA Illumina sequencing results; Table S3: Sequences of identified miRNAs present in libraries across more than one treatment; Table S4: Gene ontology (GO) enrichment analysis of targets that were predicted from upregulated miRNAs in CaCV-infected plants grown at high temperature compared to CaCV-infected plants grown at ambient temperature; Figure S5: Northern hybridizations showing the abundance of miR164 in mock or CaCV-infected capsicum plants grown at ambient temperature (AM and AV) or high temperature (HM and HV); Table S5: GO enrichment analysis of targets that were predicted from downregulated miRNAs in CaCV-infected plants grown at high temperature compared to CaCV-infected plants grown at ambient temperature; Table S6: GO enrichment analysis of targets that were predicted from upregulated miRNAs in CaCV-infected plants grown at high temperature compared to mock-inoculated plants grown at high temperature; Table S7: GO enrichment analysis of targets that were predicted from downregulated miRNAs in CaCV-infected plants grown at high temperature compared to mock-inoculated plants grown at high temperature; Table S8: GO enrichment analysis of targets that were predicted from downregulated miRNAs in CaCV-infected plants grown at ambient temperature compared to mock-inoculated plants grown at ambient temperature; Table S9: GO enrichment analysis of targets that were predicted from upregulated miRNAs in mock-infected plants grown at high temperature compared to mock-inoculated plants grown at ambient temperature; Table S10: Degradome-based miRNAs-targets analysis.
