# Supplementary material for: Involvement of MicroRNAs in the Hypersensitive Response of Capsicum Plants to the Capsicum Chlorosis Virus at Elevated Temperatures
Source: Pathogens. 2024 Aug 31;13(9):745. doi: 10.3390/pathogens13090745 (PMC11434723; doi:10.3390/pathogens13090745)
Supplement: Supplementary file 1 [file pathogens-13-00745-s001.zip › Supplementary Figures300824.pptx]

## Slide 1
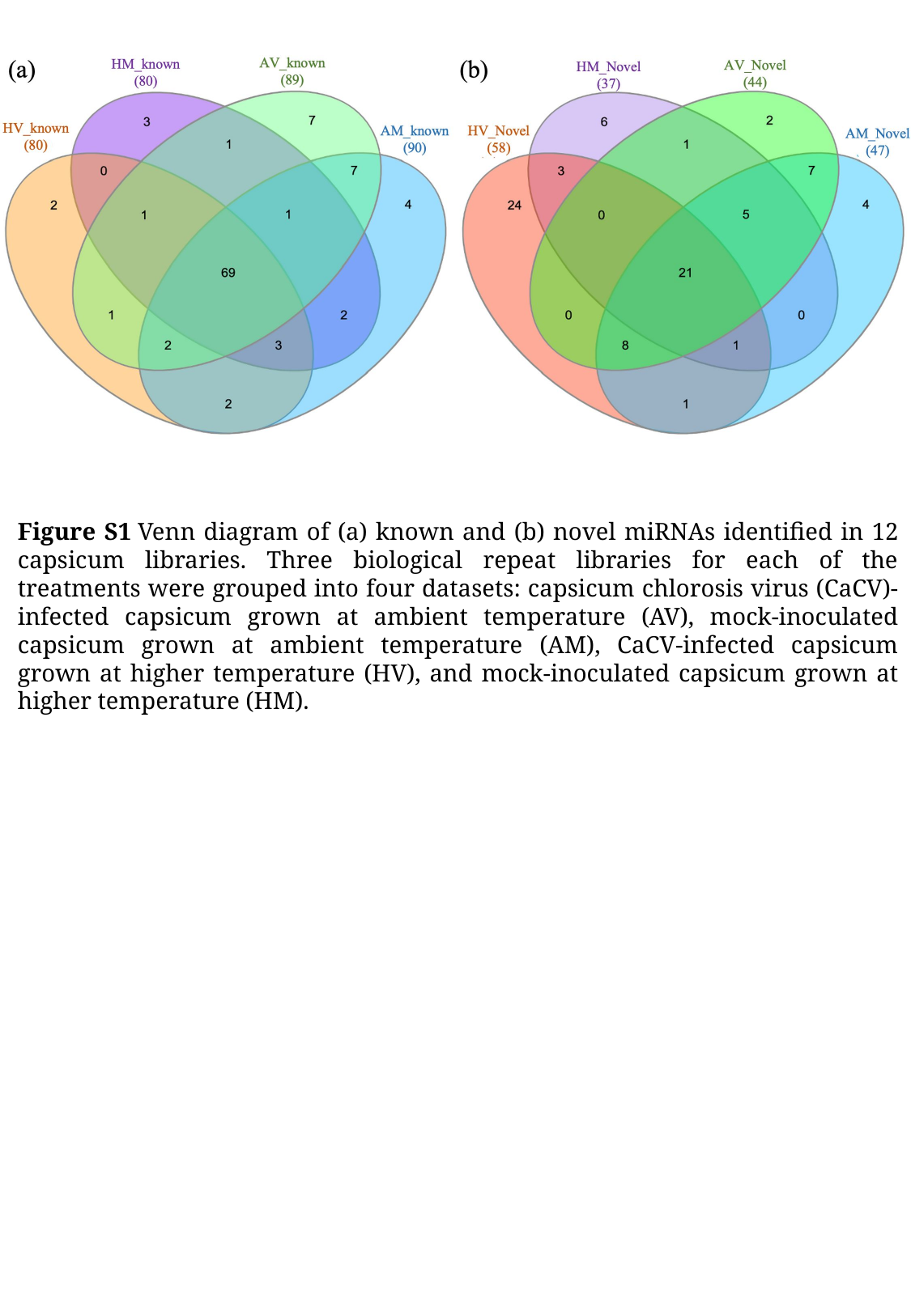

Figure S1 Venn diagram of (a) known and (b) novel miRNAs identified in 12 capsicum libraries. Three biological repeat libraries for each of the treatments were grouped into four datasets: capsicum chlorosis virus (CaCV)-infected capsicum grown at ambient temperature (AV), mock-inoculated capsicum grown at ambient temperature (AM), CaCV-infected capsicum grown at higher temperature (HV), and mock-inoculated capsicum grown at higher temperature (HM).

## Slide 2
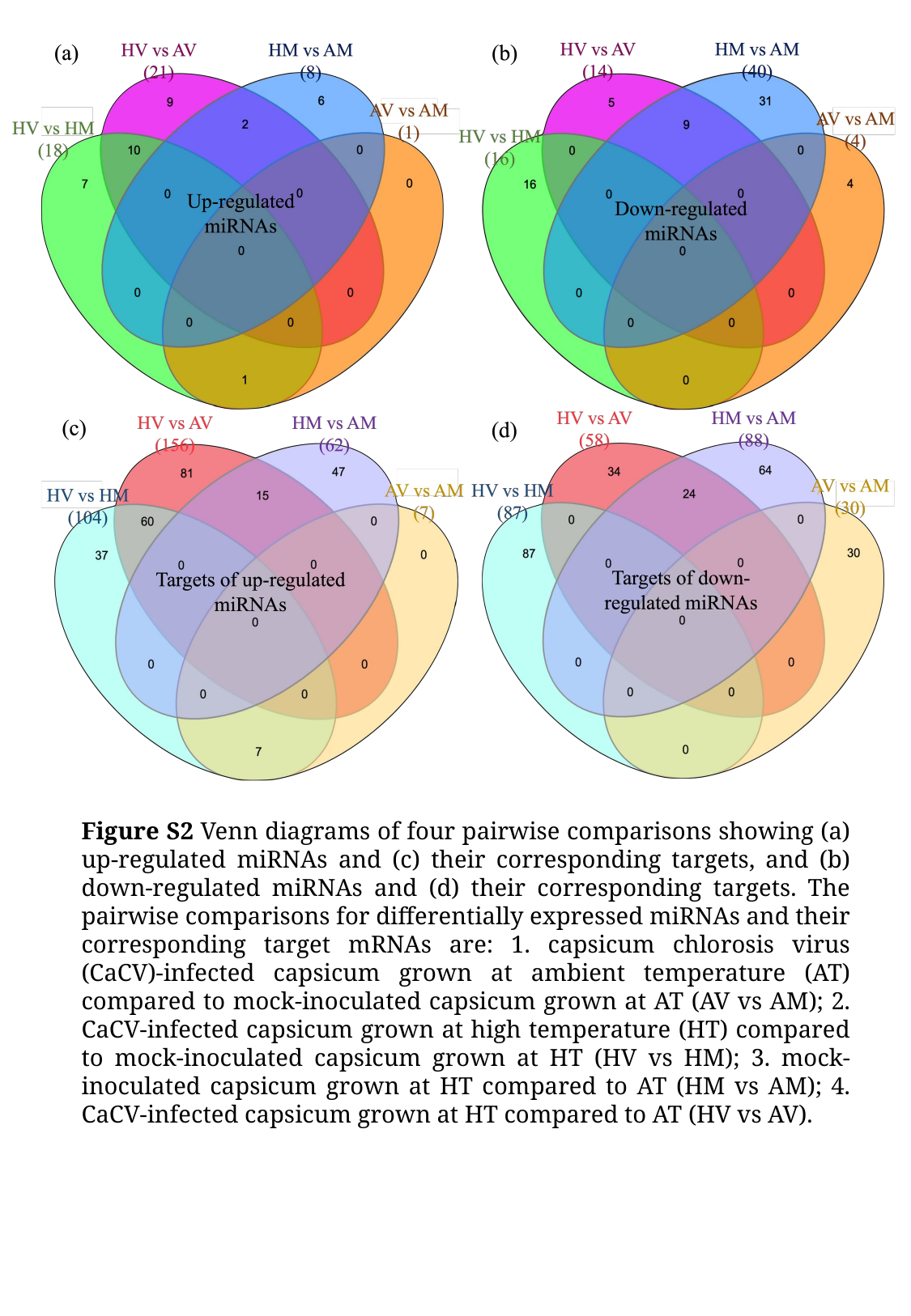

Figure S2 Venn diagrams of four pairwise comparisons showing (a) up-regulated miRNAs and (c) their corresponding targets, and (b) down-regulated miRNAs and (d) their corresponding targets. The pairwise comparisons for differentially expressed miRNAs and their corresponding target mRNAs are: 1. capsicum chlorosis virus (CaCV)-infected capsicum grown at ambient temperature (AT) compared to mock-inoculated capsicum grown at AT (AV vs AM); 2. CaCV-infected capsicum grown at high temperature (HT) compared to mock-inoculated capsicum grown at HT (HV vs HM); 3. mock-inoculated capsicum grown at HT compared to AT (HM vs AM); 4. CaCV-infected capsicum grown at HT compared to AT (HV vs AV).

## Slide 3
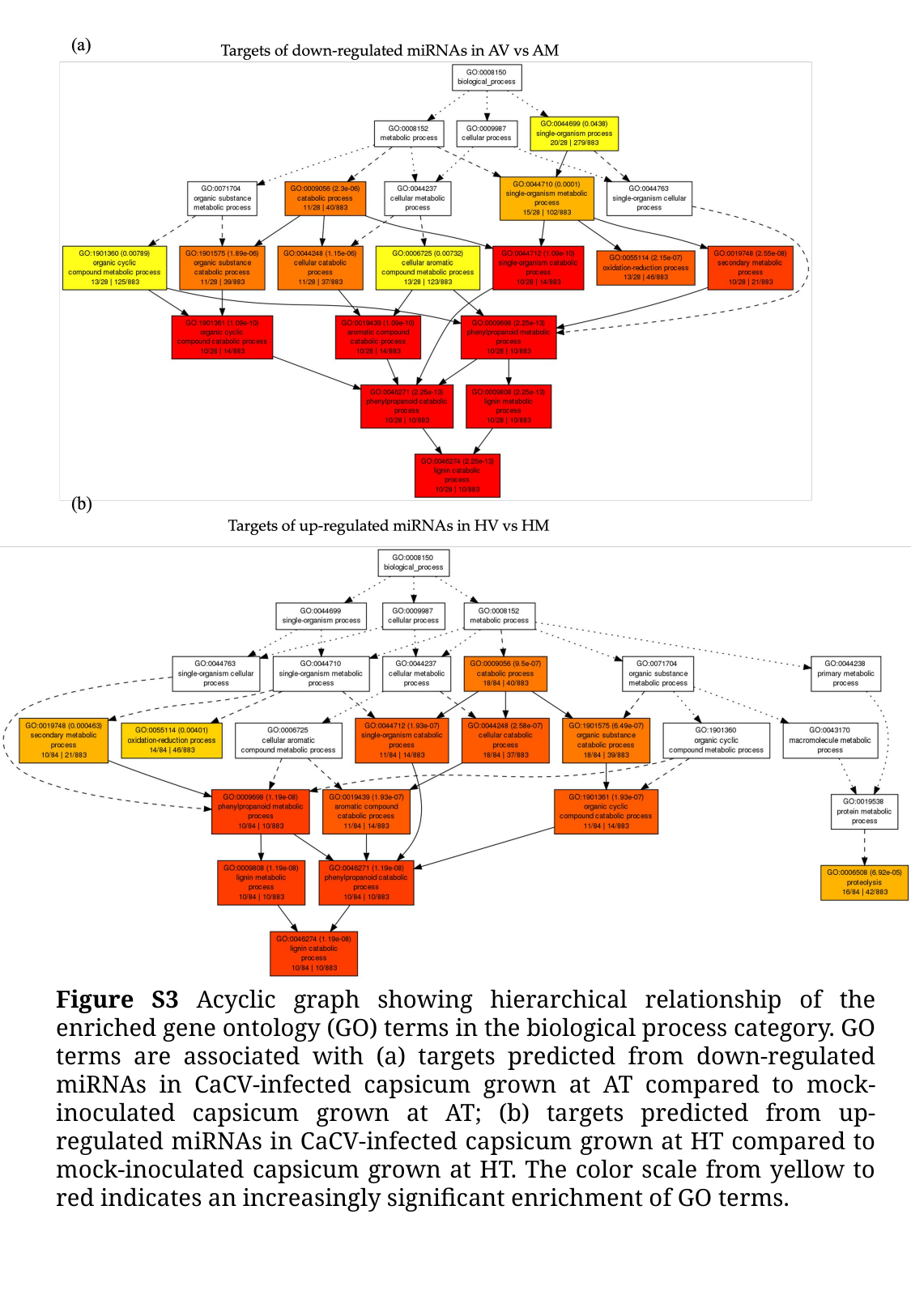

Figure S3 Acyclic graph showing hierarchical relationship of the enriched gene ontology (GO) terms in the biological process category. GO terms are associated with (a) targets predicted from down-regulated miRNAs in CaCV-infected capsicum grown at AT compared to mock-inoculated capsicum grown at AT; (b) targets predicted from up-regulated miRNAs in CaCV-infected capsicum grown at HT compared to mock-inoculated capsicum grown at HT. The color scale from yellow to red indicates an increasingly significant enrichment of GO terms.

## Slide 4
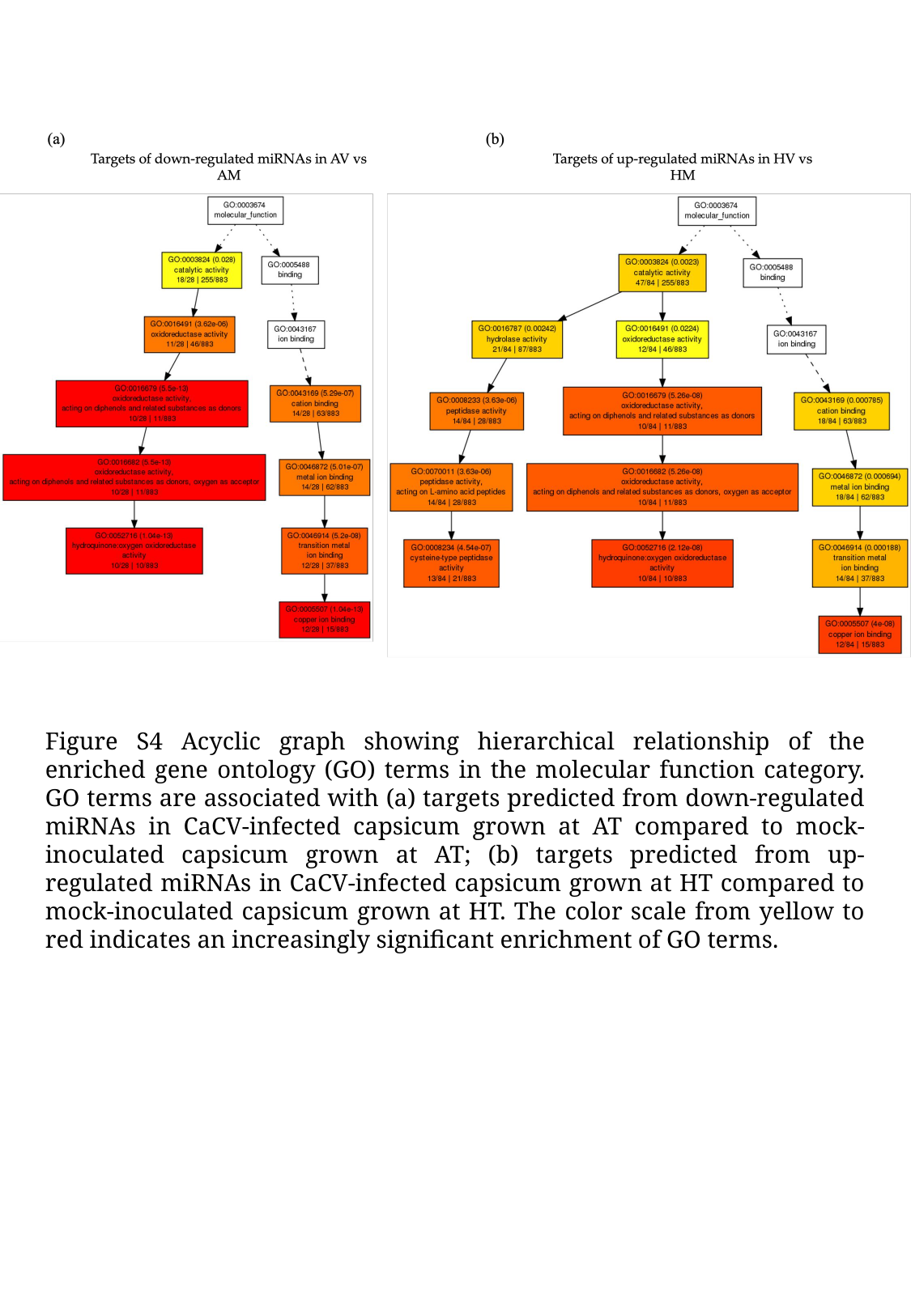

Figure S4 Acyclic graph showing hierarchical relationship of the enriched gene ontology (GO) terms in the molecular function category. GO terms are associated with (a) targets predicted from down-regulated miRNAs in CaCV-infected capsicum grown at AT compared to mock-inoculated capsicum grown at AT; (b) targets predicted from up-regulated miRNAs in CaCV-infected capsicum grown at HT compared to mock-inoculated capsicum grown at HT. The color scale from yellow to red indicates an increasingly significant enrichment of GO terms.

## Slide 5
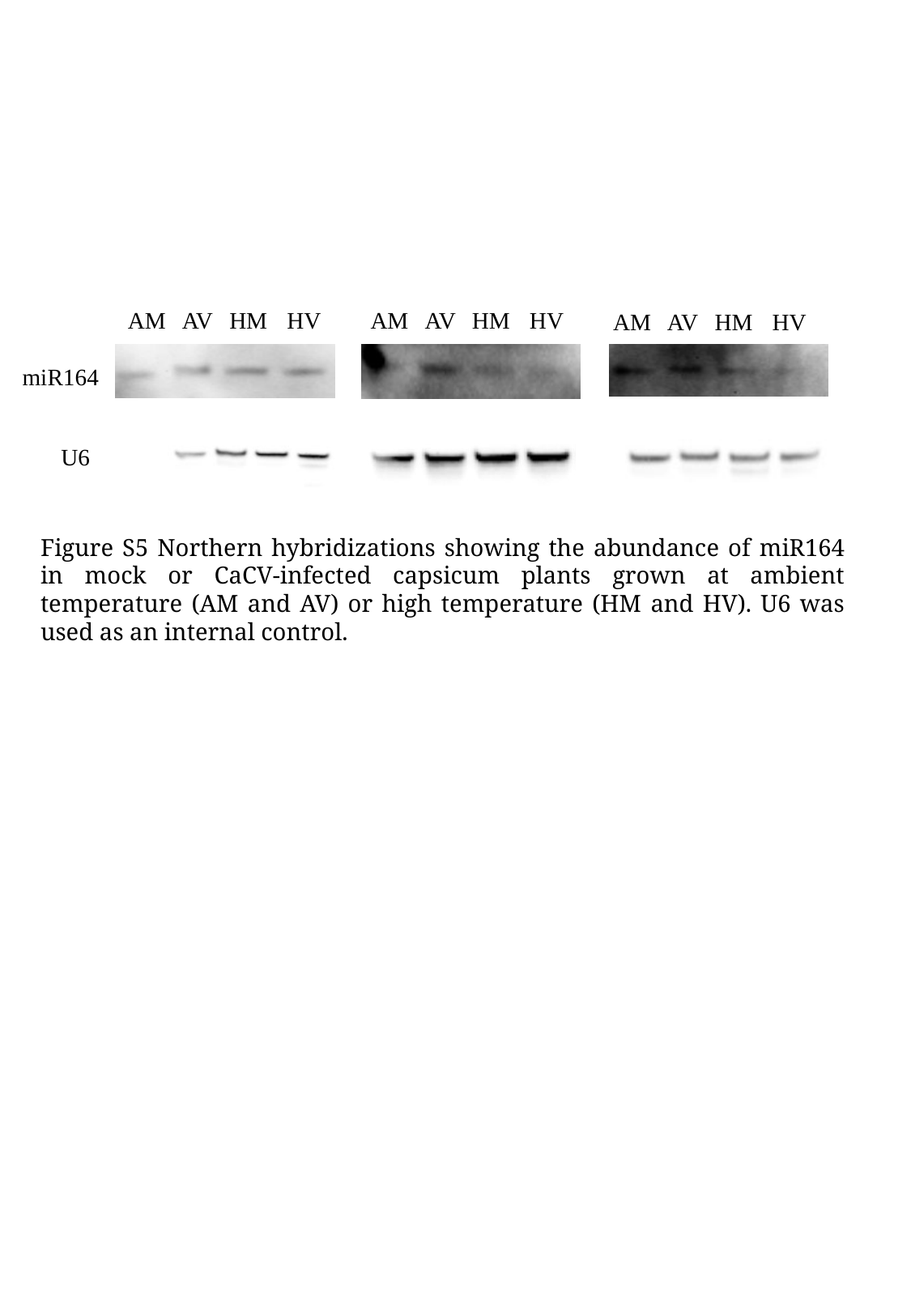

AM
HM
AV
HV
AM
HM
AV
HV
AM
HM
AV
HV
miR164
U6
Figure S5 Northern hybridizations showing the abundance of miR164 in mock or CaCV-infected capsicum plants grown at ambient temperature (AM and AV) or high temperature (HM and HV). U6 was used as an internal control.
